# Motivational and evolutionary aspects of a physical exercise training program: a longitudinal study

**DOI:** 10.3389/fpsyg.2015.00648

**Published:** 2015-05-18

**Authors:** João P. P. Rosa, Altay A. L. de Souza, Giscard H. O. de Lima, Dayane F. Rodrigues, Valdir de Aquino Lemos, Eduardo da Silva Alves, Sergio Tufik, Marco T. de Mello

**Affiliations:** ^1^Department of Psychobiology, Universidade Federal de São PauloSão Paulo, Brazil; ^2^Centro de Estudos em Psicobiologia e ExercícioSão Paulo, Brazil; ^3^Sport Psychology Laboratory, Universidade Federal de Minas Gerais, Belo HorizonteBrazil; ^4^Center for Communication and Cognitive Science (4C), Escola de Comunicações e Artes, Universidade de São PauloSão Paulo, Brazil

**Keywords:** exercise motivation, adherence, health promotion, sociability, evolutionary aspects of physical exercise

## Abstract

Several studies have indicated that motivational level and prior expectations influence one’s commitment to physical activity. Moreover, these aspects are not properly described in terms of proximal (SDT, Self Determination Theory) and distal (evolutionary) explanations in the literature. This paper aims to verify if level of motivation (BREQ-2, Behavioral Regulation in Exercise Questionnaire-2) and expectations regarding regular physical exercise (IMPRAF-54) before starting a 1-year exercise program could determine likelihood of completion. Ninety-four volunteers (53 women) included a completed protocol group (CPG; *n* = 21) and drop-out group (*n* = 73). The IMPRAF-54 scale was used to assess six different expectations associated with physical activity, and the BREQ-2 inventory was used to assess the level of motivation in five steps (from amotivation to intrinsic motivation). Both questionnaires were assessed before starting a regular exercise program. The CPG group presented higher sociability and lower pleasure scores according to IMPRAF-54 domains. A logistic regression analysis showed that a one-point increment on sociability score increased the chance of completing the program by 10%, and the same one-point increment on pleasure score reduced the chance of completing the protocol by 16%. ROC curves were also calculated to establish IMPRAF-54 cutoffs for adherence (Sociability – 18.5 points – 81% sensibility/50% specificity) and dropout (Pleasure – 25.5 points – 86% sensibility/20% specificity) of the exercise protocol. Our results indicate that an expectation of social interaction was a positive factor in predicting adherence to exercise. Grounded in SDT and its innate needs (competence, autonomy, relatedness), physical exercise is not an end; it is a means to achieve autonomy and self-cohesion. The association of physical activity with social practices, as occurs in hunter-gathering groups, can engage people to be physically active and can provide better results in adherence exercise programs for the general population.

## Introduction

Physical exercise is an effective approach in health promotion (Gill et al., 2002; Gulati et al., 2003; Greene et al., 2009), wherein the physical conditioning of those who regularly participate in physical activities increases and/or improves both their physical and psychological capacities (Cress et al., 2005; Cyarto et al., 2006). People in technologically developed societies understand the benefits of physical exercise as a healthy behavior (Crombie et al., 2004), yet they have amongst the highest inactivity rates (Dumith et al., 2011). This paradox is revealing of a strong, yet poorly characterized mechanism underlying the current lack of motivation and engagement in physical activity (Dishman, 1994; Sallis and Owen, 1999; Wilson and Brookfield, 2009).

As many as 50% of people who start an exercise program will drop out during the first 6 months (Wilson and Brookfield, 2009). Eight weeks are needed for an initiate exerciser to become a regular exerciser, but even after 6 months, the motivation of initiating exercisers remains significantly lower than long-term regular exercisers (Rodgers et al., 2010). Low levels of motivation and self-efficacy, time-shortage, low familiarity with exercise, and poor social and cultural support are considered the primary reasons why individuals fail to adhere to physical exercise programs after they begin (Sherwood and Jeffery, 2000).

A study by Brière et al. (2003), found many individuals to believe that exercise will not bring any benefit or that they are not able to perform exercise satisfactorily. This raises questions as to role and durability of existing rational arguments about the benefits of being physically active in changing the behavior of inactive individuals.

A number of theories have been proposed to explain the features of human motivation. Described by Deci and Ryan (1985), Self Determination Theory (SDT) is the most widely used theory that explains the types and levels of motivation that are associated with initiation and maintenance of behavioral changes that reflect attitudinal responses. SDT assumes that individuals have a predisposition to be active and to move their lives in desired and specific directions, rather than being passive and completely subjected to environmental forces (Vansteenkiste et al., 2012).

Self Determination Theory is an approach that considers the nature of the organism’s interaction in its environment (Matson, 2009). This framework is based on the concept of “need” (Murray, 1938; Hull, 1943). By Murray’s (1938) definition, a need is *“construct that stands for a force in the brain region, a force that organizes perception, apperception, intellection, conation, and action in such a way as to transform in a certain direction an existing unsatisfying situation.”*

Amotivation, extrinsic, and intrinsic motivation are the three main motivational stages on SDT related with the commitment level toward the activity. If SDT is applied to physical activity, the subjacent principle is that the individual can be both extrinsically and intrinsically motivated in a top–down process (Vansteenkiste et al., 2012).

In the highest level of motivation (intrinsic motivation), the subject begins the activity willingly in order to understand it, explore it, and deepen its practice. This behavior is commonly associated with feelings and sensations of interest, psychological well-being and joy (Brière et al., 2003). A systematic review of SDT and physical activity (Teixeira et al., 2012) indicated that all forms of autonomous regulation predict exercise participation across a range of samples and settings. Another conclusion is that more intrinsic participation motives or goals associated with exercise, such as affiliation and social engagement, challenge, and skill development, are clearly associated with greater exercise participation.

According to Ryan and Deci (2000), extrinsic motivation occurs if an activity is performed for a purpose other than the task itself. The reasons may vary in relation to the individual’s degree of autonomy, creating three categories – external regulation, internalized regulation, and identified regulation. Finally, amotivation is a motivational condition observed in individuals who are not adequately able to identify a good reason to perform any physical activity.

Due to its focus on the organism, its basic needs, and their fulfillment, SDT explicitly considers the primary role of self-interest in adaptive human functioning. An important issue that inhibits the development of a unified motivational theory about adherence to physical activity is the lack of literature on the synergy between proximal and evolutionary explanations.

A broader perspective would be the first step in a conceptual investigation to explore the psychological processes underlying adherence to physical activity. A focus only on outward indices of “adherence to physical activity” and its determinants such friendship quality (Duncan, 1993), popularity (Harland et al., 1999), or social skills (Schwarzer et al., 2008), can entangle the researcher in the proximate manifestations of adherence to physical activity instead of exploring the foundations of competence to adherence.

In a comparative perspective, we have come to believe that during human evolutionary history, physical activity was the salient feature of the lifestyle. Our ancestors were physically active not because they enjoyed exercise but because they needed to exercise. Early humans’ lifestyle demanded engagement in many energy-burning behaviors (Jasienska, 2013). The style of hunt-gathering life was composed of numerous physical activities (running to capture wounded prey, transporting children, building shelters) and social activities (visits to neighboring camps, dances as part of religious ceremonies or for recreation; Ruff, 2000).

As a response to this active lifestyle, an increase in aerobic and physical capacity allowed access to new food resources, influencing the development of brain components and consequently cognitive improvement in human. Recent work (Raichlen and Polk, 2013) notes that physical exercise associated with subsistence was associated with brain size and cognitive function during human evolution, increasing neurotrophins and growth factors that led to brain development. This leads to, for example, a strong selection to running long distances in humans.

Bramble and Lieberman (2004) argue that our genus, *Homo*, evolved from more ape-like human ancestors, *Australopithecus*, 2 million or more years ago because natural selection favored the survival of australopithecines that could run and, over time, favored the perpetuation of human anatomical features that made long-distance running possible. Primate species with short toes are possibly best runners than those with longer toes, indicating an evolutionary adaptation to humans as good runners (Rolian et al., 2009). Another study shows that the *gluteus maximus*, muscle is also an adaptation to long-distance running, together with long legs and big buttocks in comparison with other primates. The reorganization of leg muscle anatomy in response to selective pressures on the environment over time was important to human pelvis development and bipedalism in order to increase the payoffs of foraging (Lieberman et al., 2006).

An evolutionary approach to motivation indicates that humans have many motivational mechanisms to specific information processing characteristics, and a cultural environment influenced by social and physical factors that frames this behavior. In the absence of an overpowering extrinsic motivation (for example, need to gather food), modern man must rely on other forms of motivation to initiate and maintain a regular exercise program. Regular physical exercise can be effective in yielding positive effects on health and quality of life. However, most people do not gain the benefits provided by regular physical exercise because they drop out of these exercises early.

Our hypothesis is that some levels or forms of motivations at the outset are better predictive of long-term adherence to an exercise program. Moreover, we expect that *a priori* expectations to physical activity can be a drive to increase (or reduce) the level of motivation of participants to be adherent to physical activity programs. In an evolutionary perspective, we hypothesize that physical activity alone is not source of intrinsic motivation. To be active is mainly associated with collective/social practices used as ways to achieve important resources or mating. Socialization and integration of physical activity on the daily life are the main expectations to increase the initial adherence to physical activity program by the authors.

Using a broader approach to explain our findings, the present study aims to determine whether the level of motivation through SDT [using BREQ questionnaire and its Relative Autonomy Index (RAI)] and expectations related to physical activities (using IMPRAF-54 questionnaire) before starting the long-term exercise protocol can determine the participant’s chance of completing the exercise protocol.

## Materials and Methods

### Participants

Ninety-four volunteers were invited to participate via personal contact and published media. All volunteers were older than age 18 and had completed at least 8 years of schooling. Volunteers with any previous history of neurological disease, who already regularly practiced physical exercise during the 6 months (or already practiced any kind of physical activity in high performance during lifetime) prior to the start of the study or who had any history of alcohol or illegal drug abuse were excluded. Prior to being included in the study, all volunteers were informed regarding the study procedures and any potential discomfort or risk associated with the assessment process. They were also required to provide written informed consent by signing consent forms. All procedures that were used in the present study complied with the Declaration of Helsinki (1975), and the study was approved by the Research Ethics Committee at the Universidade Federal de São Paulo – UNIFESP on 08/02/2011, protocol number 0155/11.

### Design and Procedure

Participants performed a 1-year exercise program that consisted of three sessions per week for a period not exceeding 90 min per session. They chose the type of physical exercise they would like to perform (aerobic training, strength training, or concurrent training), and a period of familiarization with the physical exercise protocol was allowed. A participant was considered a drop-out if he or she did not attend the training for two consecutive weeks or 36 non-consecutive training sessions (25% of the study protocol).

Before the participants started the training protocol, *a* Bod Pod^®^ device (Life Measurement, Inc., United States) was used to establish their body mass index (BMI; in kg/m^2^) and body fat. During the assessment, the following variables were measured: density (kg/L), total body mass (kg), lean mass (kg), fat mass (kg), percentage (%) of lean mass, and percentage (%) of fat mass. The heights of the participants were measured directly using a stadiometer (Holtain Ltd., Crymych, Dyfed, UK).

The level of motivation to begin a program of physical exercise was assessed by a translated version of the Behavioral Regulation in Exercise Questionnaire–2 (BREQ-2; Markland and Tobin, 2004), which was administered to each participant. This version has been translated to and validated in Portuguese language (Palmeira et al., 2007).

The BREQ-2 inventory comprises 19 Likert items, each of which has five possible answers that are scored on a scale of 0–4 (0 = Not true for me; 4 = Very true for me). The questionnaire assesses five constructs: *amotivation* – e.g., “I think that exercising is a waste of time”; *external regulation* – e.g., “I exercise because other people tell me I should”; *introjected regulation* – e.g., “I feel guilty when I do not exercise”; *identified regulation* – e.g., “I value the benefits/advantages of exercising”; and *intrinsic motivation* – e.g., “I enjoy my exercise sessions.”

As with other measures of the continuum of self-determination, the BREQ-2 can be used either as a multidimensional instrument that provides separate scores for each subscale or as a unidimensional index of the degree of self-determination, known as the RAI (Ryan and Connell, 1989). RAI describes the level of autonomous behavior: a higher RAI is associated with a more autonomous participant and a lower RAI, a non-autonomous participant.

The Inventory of Motivation for the Regular Practice of Physical Activity and/or Sports (IMPRAF-54) was used to assess the motivational dimensions associated with the practicing exercise regularly. This instrument comprises 54 items that are clustered according to the particular dimension of motivation that is being assessed: *stress control* (e.g., releasing mental tension), *health* (e.g., maintaining physical fitness), *sociability* (e.g., spending time with friends), *competitiveness* (e.g., winning a competition), *esthetic* (e.g., having an attractive appearance), and *pleasure* (e.g., personal enjoyment).

The participants’ responses to the IMPRAF-54 items were obtained using five-point Likert scales that ranged from 1 to 5. A score of 1 meant “this motivates me very little,” and 5 meant, “this motivates me a great deal.” Each dimension was assessed using the same number of questions. The reliability and validity of this instrument were previously tested and verified (Barbosa and Balbinotti, 2006).

The group allocation is described in **Table 1**. The volunteers were separated into two groups (CPG, completed protocol group and DG, drop-out group) based on his attendance in the 1-year protocol, yielding the subsequent analysis.

**Table 1:** Characteristics of the 94 study participants (Mean ± SD).

		Completed protocol group	Drop-out group	*F*	*p*
		(CPG) *n* = 21	(DG) *n* = 73		
Gender	Male	8	33		
	Female	13	40		

Age (years)	34.03 ± 9.71	39.14 ± 10.09	32.56 ± 8.34	9.209	0.001*
Weight (kg)	73.14 ± 14.63	70.25 ± 11.85	73.97 ± 15.31	1.05	0.31
Height (cm)	168.07 ± 9.41	167.61 ± 8.77	168.20 ± 9.64	0.062	0.8
BMI (kg/m^2^)	25.79 ± 4.23	24.94 ± 3.51	26.04 ± 4.41	1.093	0.3
BF (%)	29.37 ± 9.51	30.1 ± 10.93	29.16 ± 9.13	0.155	0.69
**BREQ-2 Motivational levels**
Amotivation		0.38 ± 0.57	0.41 ± 0.57	0.056	0.81
External regulation		0.58 ± 0.66	0.47 ± 071	0.412	0.52
Introjected regulation		1.67 ± 1.04	1.66 ± 1.03	0.001	0.99
Identified regulation		2.88 ± 0.82	2.99 ± 0.65	0.38	0.54
Intrinsic regulation		2.89 ± 0.91	2.88 ± 0.89	0.003	0.95
RAI		10.46 ± 4.65	10.76 ± 4.62	0.071	0.79
**IMPRAF-54 -Motivation for regular practice of physical activity**
Stress control		28.29 ± 5.75	26.04 ± 7.24	1.704	0.19
Health		34.19 ± 4.18	33.84 ± 4.11	0.012	0.73
Sociability		24.48 ± 7.26	20.10 ± 8.16	4.918	0.03*
Competitiveness		13.67 ± 6.53	12.73 ± 6.88	0.311	0.58
Esthetic		31.00 ± 5.10	30.47 ± 8.93	0.068	0.79
Pleasure		31.38 ± 4.75	33.63 ± 8.67	1.294	0.26

BMI, Body Mass Index; BF, Body Fat; RAI, Relative Autonomy Index; *p < 0.05 (ANOVA).

### Statistical Analysis

Descriptive data were presented as the mean and SD. One-way ANOVA was used to verify initial differences between the CPG and DG. A 2 (CPG and DG) × 3 (settings of the BREQ-2: identified, introjected and intrinsic) two-way ANCOVA was performed by assessing the results of the IMPRAF-54 and scores with the Sidak *post hoc* test. These results were controlled for age and BMI of the participants and the prerequisites for these analyzes were verified (homogeneity of variances among groups).

To investigate the associations between adherence and motivational level better, a binary logistic regression model was constructed. Adherence to training (1-CPG 0-DG) was the dependent variable, and IMPRAF-54 score (stress control, health, sociability, esthetic, and pleasure) and level of self-determination according to BREQ-2 score (amotivation, external regulation, introjected regulation, identified regulation, and intrinsic motivation) were associated factors. The regression model yielded a categorical variable that represents the motivational level prior to physical exercise for each participant. Finally, based on logistic regression and the factors associated with adherence to training, ROC curves were calculated for the IMPRAF-54 scores that reached statistical significance in the previous tests. The analyses were performed using SPSS software (v.19), and the level of significance was established at *p* < 0.05.

## Results

Seventy-three volunteers (77.6% of the entire sample – 40 women) did not complete the protocol, and 21 volunteers (13 women) completed the full 1-year exercise protocol. No differences were found on IMPRAF-54 domains and BREQ-2 between the men and women participants.

**Table 1** shows the descriptive data of the participants, including demographic information, physical variables and the results of questionnaires. Also shown are the ANOVA results, which revealed a significant effect of adherence group in the Sociability score of the IMPRAF-54. The group that completed the training (CPG) had a higher Sociability means (CPG – 24.4 ± 7.2 vs. DG – 20.1 ± 8.1, *p* = 0.03). Furthermore, we observed that people with a higher mean age completed the training more frequently (CPG – 39.9 ± 10.1 vs. DG – 32.5 ± 8.0, *p* = 0.001).

**FIGURE 1:**
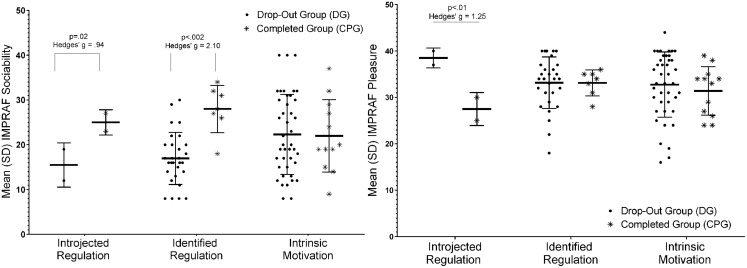
**Results of a two-way ANCOVA to evaluate the effect of adherence (CPG, Completed Protocol Group and DG, Drop-out Group)**. These results were controlled for age and Body Mass Index (BMI) of the participants. *p < 0.05.

Using age and BMI as covariates, we verified the difference between the CPG and DG, and the two-way ANCOVA showed a group effect [*F*_(7,80)_ = 2.12, *p* = 0.005] of the Sociability (*p* = 0.04) and Pleasure scores (*p* = 0.008) according to the IMPRAF-54 questionnaire. **Figure 1** shows that people who completed the training had higher levels of sociability and lower levels of enjoyment. These findings suggest that the participants’ adherence to the training was positively associated with the expectation of socialization before starting the exercise program and inversely associated with expectation of pleasure from exercise itself before starting the program.

Moreover, we found a significant interaction effect between adherence (CPG × DG) and level of Behavioral Regulation in Exercise (BREQ-2 groups) on the RAI [*F*_(14,160)_ = 1.33, *p* = 0.04]. The Sidak *post hoc* test showed that people with introjected motivation (6.5 ± 2.2) presented lower levels of RAI than people with intrinsic motivation (11.8 ± 0.7), but only in the CPG group (*p* = 0.03).

Additionally, a Multivariate binary logistic regression was performed. Adherence to training (CPG) was used as the dependent variable, and the Sociability and Pleasure scores on the IMPRAF-54 and BREQ-2 classification of the level of self-determination were used as independent variables. The model shows (**Table 2**) a onefold increment in the IMPRAF-54 Sociability score increased the chance of being in the CPG group by 10% (OR = 1.1 CI 95% 1.0-1.2, *p* = 0.04). However, participants who had higher expectations associated with pleasure prior to training had lower rates of adherence at the end of 1 year (OR = 0.8 CI 95% 0.7-0.9, *p* = 0.03). Each unit added to the IMPRAF-54 Pleasure score increased the chance of being in the DG group by 16%.

**Table 2:** Binary logistic regression with 95% CI Odds Ratio for 94 participants who started a 1-year exercise protocol.

Reference group (CPG)	*p*	OR	95% CI OR	
			Lower	Upper
Stress control	0.15	1.09	0.96	1.22
Health	0.92	0.99	0.82	1.19
Sociability	0.04*	1.11	1.04	1.23
Competitiveness	0.37	0.95	0.85	1.06
Esthetic	0.20	1.05	0.97	1.14
Pleasure	0.03*	0.85	0.74	0.98
Age	0.00*	1.15	1.07	1.25

*p < 0.05. These results were controlled for BMI of participants.

**FIGURE 2:**
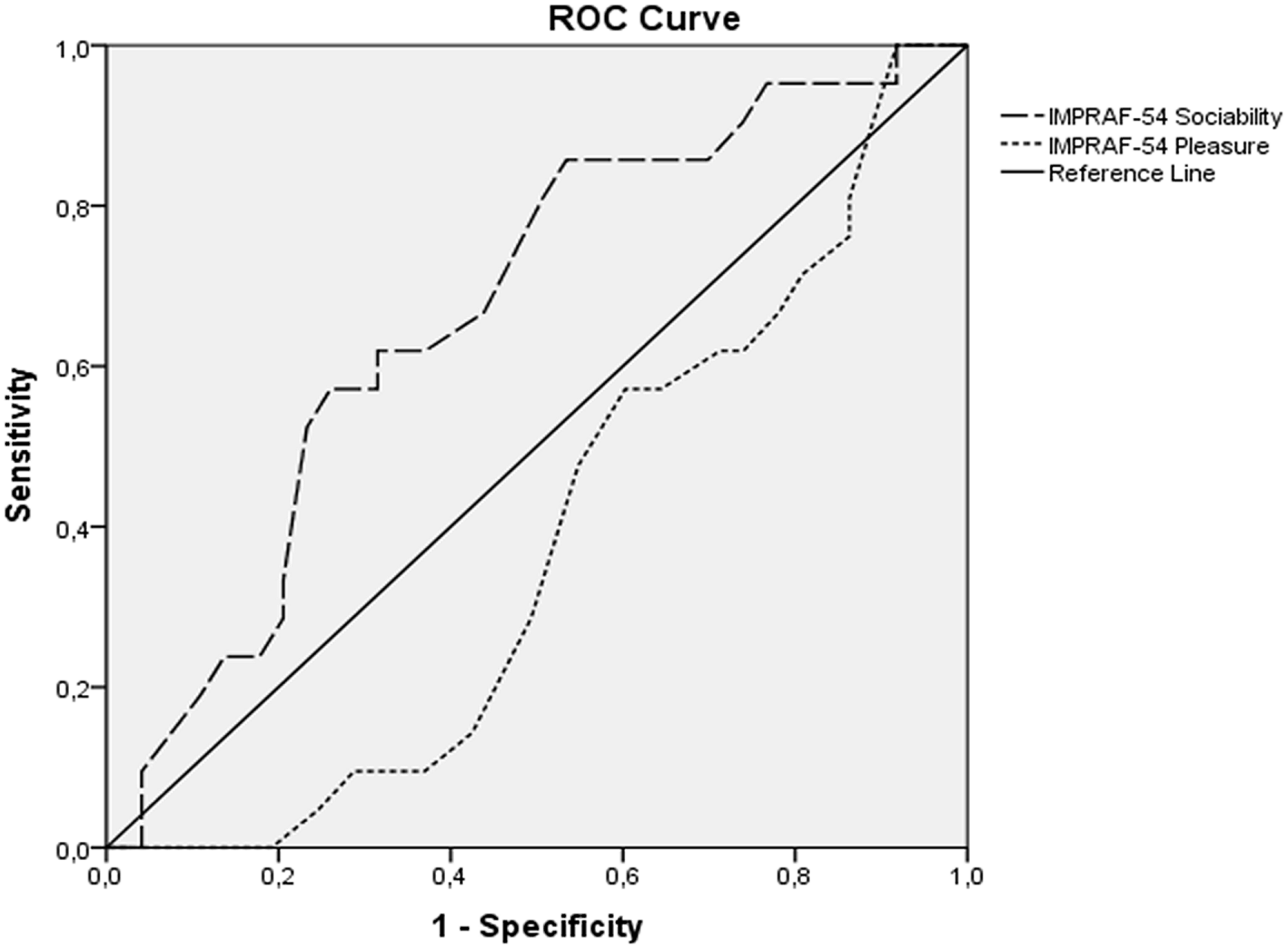
**ROC curve for Sensibility/Specificity related to IMPRAF-54 “Sociability” in the CPG and IMPRAF-54 “Pleasure” in the DG**.

Based on logistic regression and the factors associated with adherence to training, ROC curves were made for IMPRAF-54 Pleasure and Sociability scores. **Figure 2** shows that for a Sociability score of 18.5 points, there was an 81% sensitivity and 50% specificity for adherence to training (AUC 0.67 CI 95% 0.54-0.79; *p* = 0.01). For a Pleasure score of 25.5 points, the sensitivity was 86% and the specificity was 20% for non-adherence to the training protocol (AUC 0.61 CI 95% 0.50-0.73; *p* = 0.008).

## Discussion

We found that differences in *a priori* expectations that motivate people to a training program can augment or reduce the chances of completing the exercise protocol. Several studies have addressed the advantages of physical exercise to people at the behavioral (Woods et al., 2002), physiological and molecular levels (Brown et al., 2013). Nevertheless, to enjoy the benefits of exercise, people need to first commit, and the study of the conditioning factors to adherence are still scarce and undervalued.

In the present study, we considered people who did not intend to be high-performance athletes but rather who wanted to reduce their levels of physical inactivity and improve their quality of life. Elite athletes do not necessarily have motivation for training itself but for the outcome of the competition (Vallerand and Losier, 1999). Physical exercise itself is a clear modification on individual behavior but, in the long term, provides individual attitude modification about the benefits of physical activity – to not only avoid harm or diseases but also as self-esteem protection.

According to SDT, motivated individuals expend some energy to perform various tasks, and their performance is regulated by intrinsic or extrinsic motivations. However, seeking pleasure through exercise can be a source of extrinsic motivation for a given period of time, working in sporadic events (Brière et al., 2003). The possibility to find social support can more effectively regulate the decision to exercise frequently (Sherwood and Jeffery, 2000).

Based on our ROC curve results, the sociability cutoff to finish a 1-year exercise protocol was 18.5 points, which is a middling level according to the possible range for Sociability scores on IMPRAF-54 (0–40 points). This finding indicated a moderate expectation to socialize through physical practice is sufficient to significantly improve the odds for sedentary people to adhere to a 1-year exercise protocol, adding the benefits of this behavior to their lives.

Considering adherence to the physical exercise protocol (in months), those who completed the 1-year exercise program had a greater expectation to socialize even with the same level of motivation (identified regulation) compared with the dropout group. This finding agrees with some studies (Rhodes et al., 2002; Rhodes and Courneya, 2003; Hoyt et al., 2009) in which extroversion (characterized by positive emotions and a tendency to seek the company of others) can affect behavioral change with respect to exercise. Several studies (Mikel, 1983; Yates et al., 1983) have found that shy and introverted people tend to engage in individual sports, such as running, whereas others have a preference to collective sports (Dishman, 1994; Sallis and Owen, 1999). In our study, the participants were allowed to choose the type of training they felt comfortable performing. Choosing the training program instead of using a homogeneous program allowed the authors to avoid the confounder in which participants would drop out due to disliking a particularly type of training.

According to Dalle Grave et al. (2011), cognitive-behavioral strategies are needed to increase and maintain the frequency or intensity of an exercise regimen. Certain people are more attached to individual activities (such as running or weightlifting), whereas others prefer group activities (such as soccer or basketball). These dispositions can be identified by *a priori* screening tests to identify these personality strategies as a way to increase adherence to regular physical practice by population.

Even with these procedures, despite knowing the health benefits of regular exercise (Gill et al., 2002; Lochbaum and Lutz, 2005; Cyarto et al., 2006; Harvey et al., 2010), many people still eventually choose to stop exercising, in which the lack of motivation is a main reason for the low rates of adherence to exercise programs. The feelings of pleasure expected from the physical training often negatively affect the idealization of physical activity (especially in people who had never been to the gym).

Supported by evolutionary approaches (Cordain et al., 1997; Jasienska, 2013), exercise cannot be considered a primary stimulus to yield motivational and attitudinal change; it needs to be a by-product for other reinforcing social practices. Physical activity in evolutionary times was not the preferred choice but was a necessity – there is no way to obtain food other than through physical activity (Jasienska, 2013).

A study conducted by Eaton and Eaton (2003) shows that among hunter–gatherers, duration and type of activities had seasonal patterns with weekly variation on the routine. During some days of the week, people went on physically demanding hunting and gathering trips, but other days were spent in camp – eating, resting, and socializing. Inactive days were clearly preferred over days when people had to work. This preference for inactivity can be interpreted as an adaptation to reduce the metabolic expenditure (Williams and Nesse, 1991). Maybe this trait (the tendency to inactivity) is a basis for the difficulty for modern humans to start an exercise program and continue it for a long period of time (Astrand, 1986; Jasienska, 2013).

The tendency to inactivity does not mean that we are left with finding rational bases for being active, or for creating contexts which are supportive of activity. Nowadays, in modern cities, the requirement of physical activity in our routine in order to feel competent, autonomous, and related with others is smaller than in collective societies. According with SDT, these three psychological innate needs (competence, autonomy, and relatedness) serve, under appropriate conditions, to guide people toward more competent, vital, and socially integrated forms of behavior. In this sense, physical exercise by itself is not a need; it is a means to achieve innate needs.

An initial motivational level to start a physical activity program is important. In our results, participants lacking intrinsic motivation (but in introjective or introspective levels) were susceptible to their prior expectations in a positive (socialization) or negative (pleasure) way. Associate the prior expectations to exercise as a way to achieve intrinsic motivated needs according to SDT (like autonomy, self-cohesion, and attachment) can increase the level of commitment to practice on short-term and enhance the prior level of motivation to physical activity.

Self Determination Theory is a theory of the proximal causes of motivational states and processes formulated in terms of immediate social contexts, developmental histories, and individual differences. But the theory of needs (Murray, 1938; Deci and Ryan, 2000) and of human nature is consistent with the belief that distal causes of human psychological functioning lie in evolutionary history.

Despite the explanation that evokes the idea of environment of evolutionary adaptedness (EEA) according to the evolutionary psychology perspective (EP), it is easy to credit the lack of adherence to exercise in modern humans to the notion of “adaptive lag” (based on the idea that the human cognitive process evolved in response to selection pressures in ancestral conditions – EEA – and is not adaptive in a contemporary world that changed substantially in recent millennia).

According to this perspective, adaptations of the human mind should change at a slow pace and need hundreds of generations to establish (Bolhuis et al., 2011). Modern evidence supports the role of Holocene activity (last 10,000 years) affecting responses to the environment by human activity (such as adoption of agriculture, domestication of animals; Laland et al., 2010); increased human density and dietary changes (Voight et al., 2006); disease spread as a result of modern living (Barnes et al., 2010); and changing genes expressed in the human brain (Lloyd and Feldman, 2002; Voight et al., 2006).

Understanding the different levels of the adaptationist perspective and switching to the time-frame of the EEA, we can identify new applications for evolutionary perspectives, including a method to encourage people’s adherence to physical activity in modern cities.

Our findings suggest that a change in how society advertises the need for exercise is warranted. The adoption of advertising by health authorities (and gyms) to promote activity should consider that the mode of motivation provided can be optimized by adapting the people expectations and targets (time out for exercise as part of social time); establishing the difference between the terms “to be in shape” and “be active.” “To be in shape” is a state with a strong cultural influence that defines these characteristics, changing across places, and times.

For example, a professional weight lifter (with muscles and strength) or a runner (skinny with great stamina) can be accepted as a model of a “good shape.” To be in shape, people do not need to be active – this can be achieved through surgery, diet, or medicine – and many people use these solutions to obtain the results as soon as possible.

“To be active” on the other hand, implies a deep behavioral change; it is a way to have access to primary stimuli, including in-group activities and socialization, which are intrinsic motivation sources, such as in our ancestral environment. Despite the relevant differences in the !Kung-san way of living and the sedentary participants of our study, to understand culture as a by-product of Natural Selection, we have a better framework of how evolutionary theory can enrich research in physical activity and public health. Cultural practices, customs, and beliefs are some of the ways that humans have invented to address biological trade-offs.

Studies have reported the proportion of sedentary people who were exposed to collective and sporadic practices (public health exercise activities) such as “Agita Sao Paulo” in Brazil (Matsudo et al., 2003) or abroad (Kahn et al., 2002) that started an exercise program on their own. We suspect that the “conversion rate” (people who participate in a public health exercise program for a weekend and start a physical exercise routine by themselves afterward) is low, but a higher integration of physical activity associated with labor routine is an important point to reduce problems related to sedentary behavior, improve quality of life, and develop interdisciplinary studies in public health.

Finally, several limitations of our study should be addressed. Our findings suggest that the identification of motivational level and the prior expectations of regular practice before starting a training program are important, particularly considering adherence. In our results, we did not have participants who were classified in levels of amotivation and external motivation according to the BREQ-2. This approach can be explained because some minimal level of motivation is needed for the initial engagement to an exercise program, not only to start training but also to participate in this type of study. Another point is that relative satisfaction of expectations in the course of the exercise program was not followed.

Our results indicate that the expectation of social interaction was a positive factor in predicting maintenance of an exercise program. We therefore suggest that structuring physical exercise sessions will facilitate socialization and may increase adherence. These findings should be considered for the development of collective programs of physical activity for the population, improving the quality of life and reducing the incidence of problems related to physical inactivity.

## Author Contributions

This study was conducted by JR, GD, DR, VD, and ED. AD proposed the idea, performed the analyses, and helped JR to draft the manuscript. ST and MD supervised and guided the writing of the manuscript. All authors read and approved the final manuscript.

## Conflict of Interest Statement

The authors declare that the research was conducted in the absence of any commercial or financial relationships that could be construed as a potential conflict of interest.
